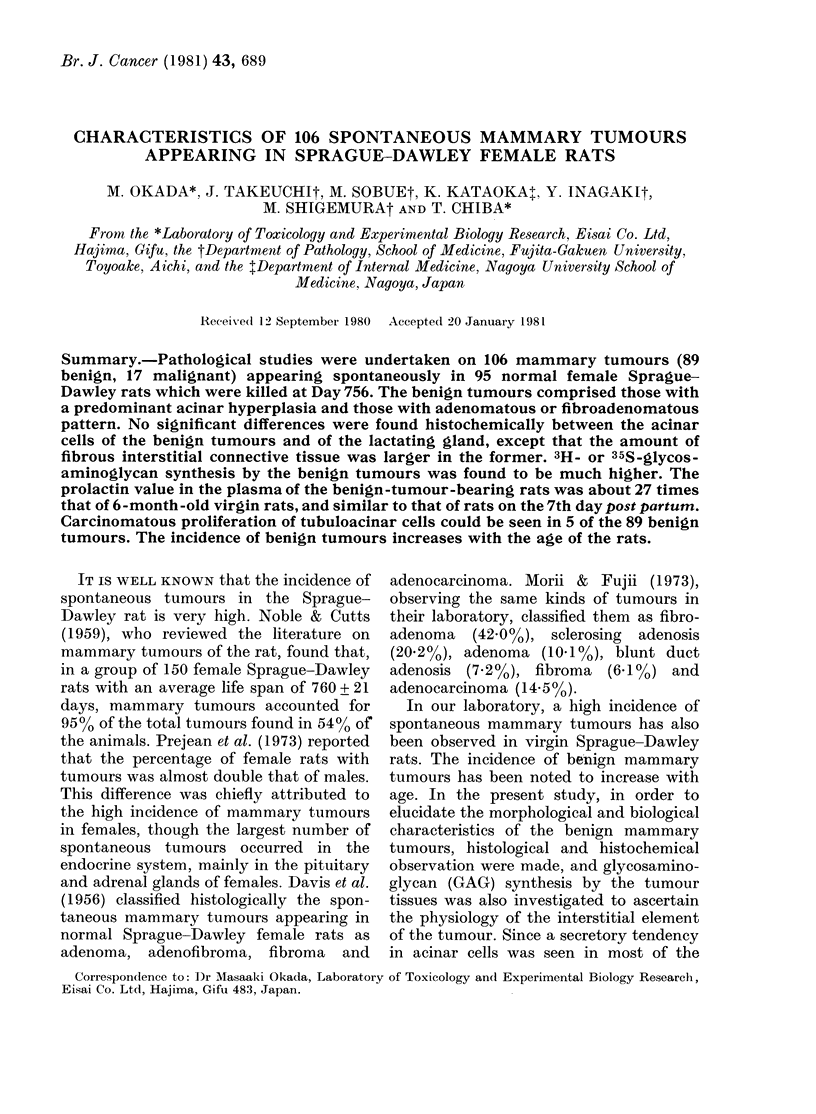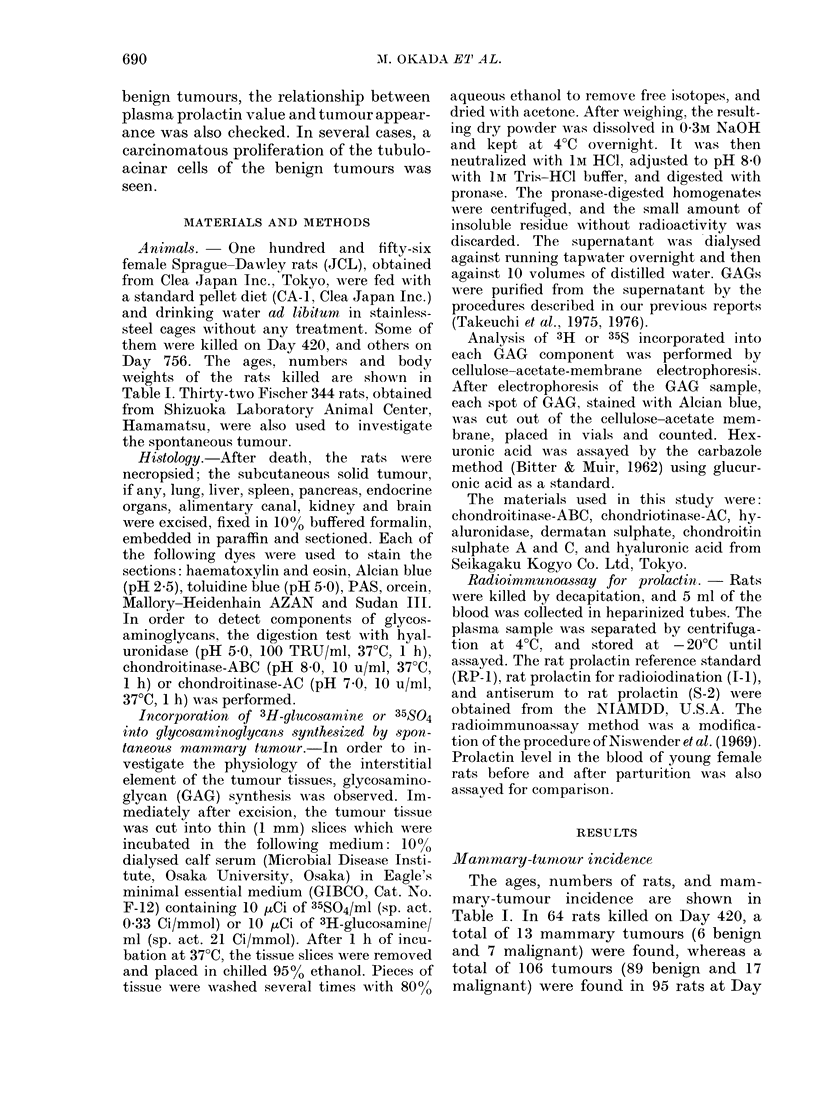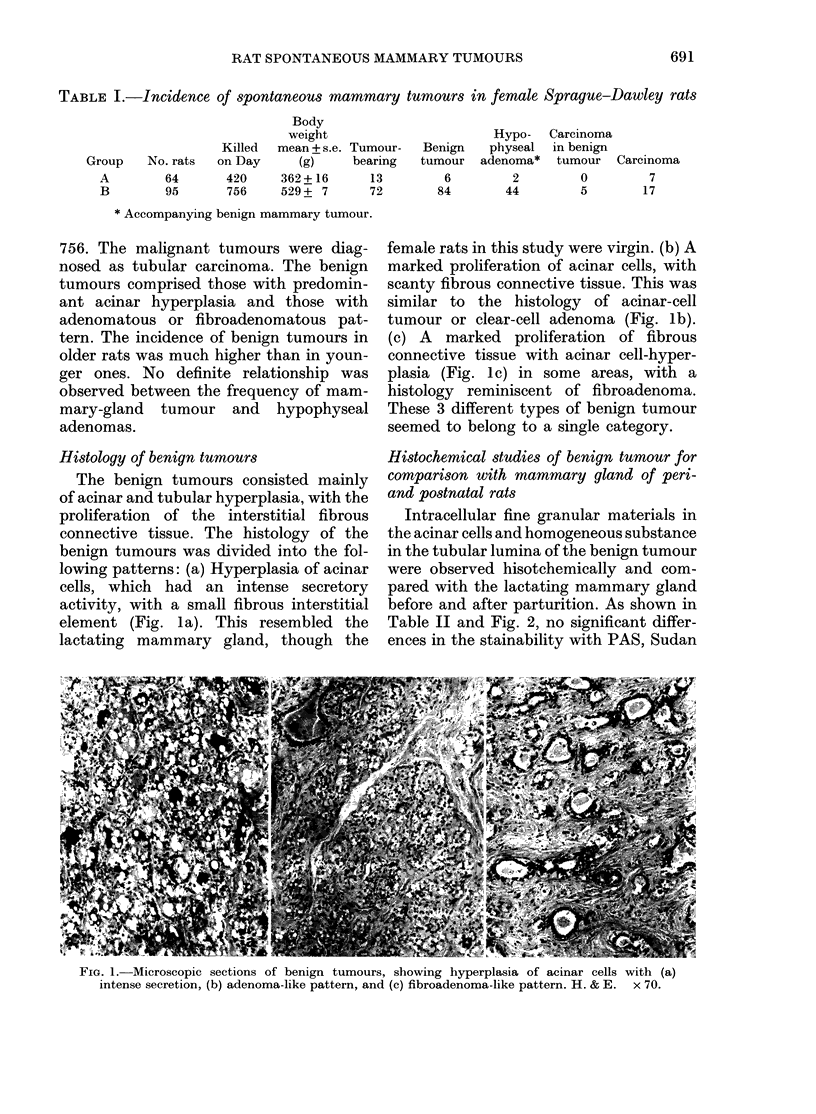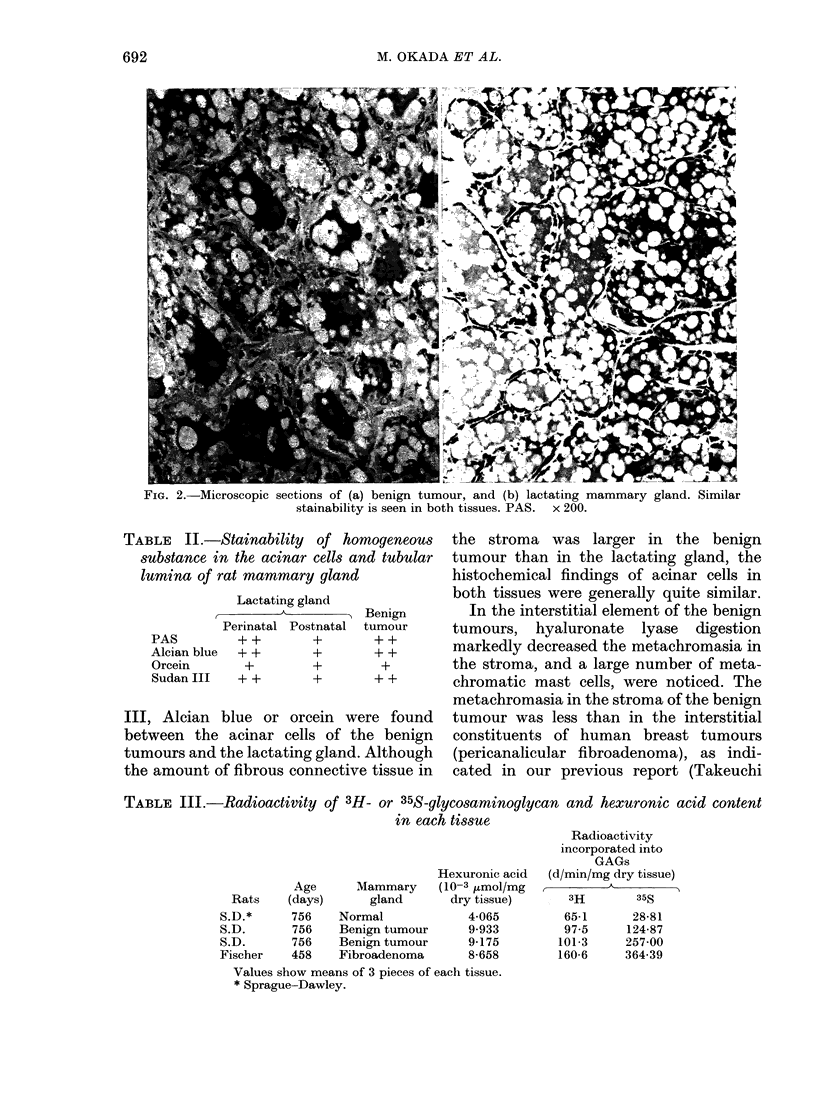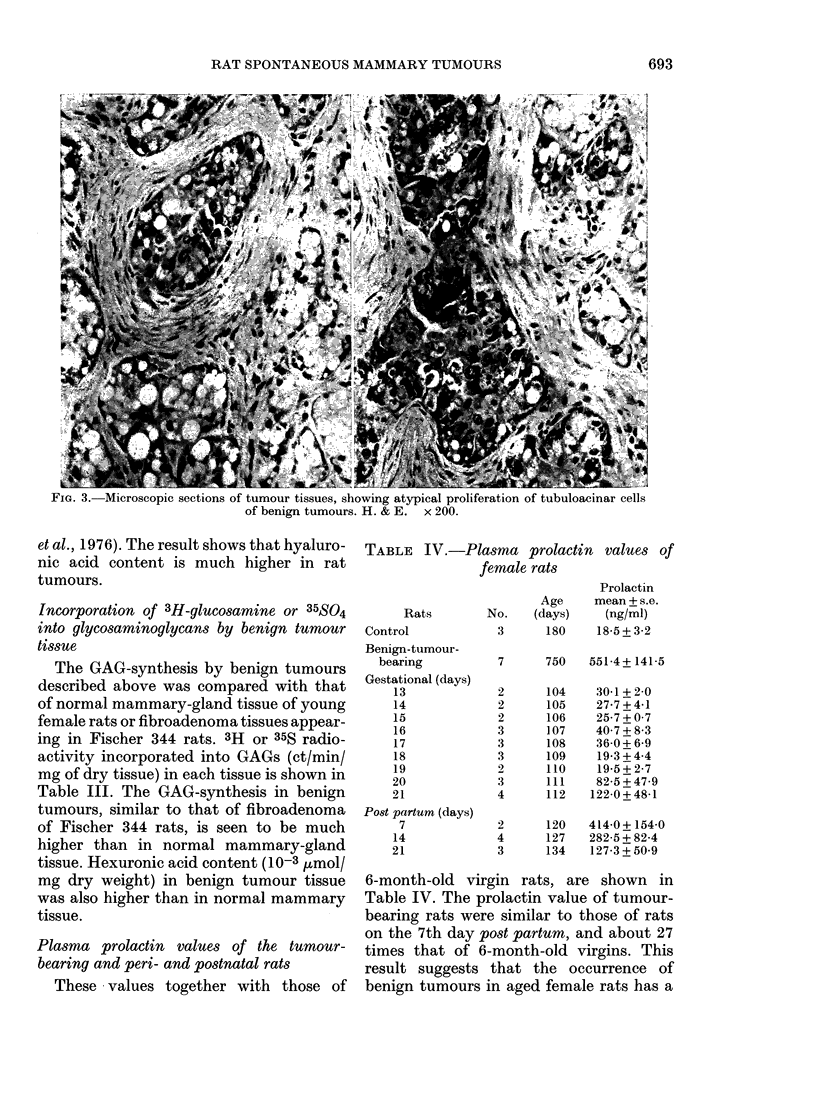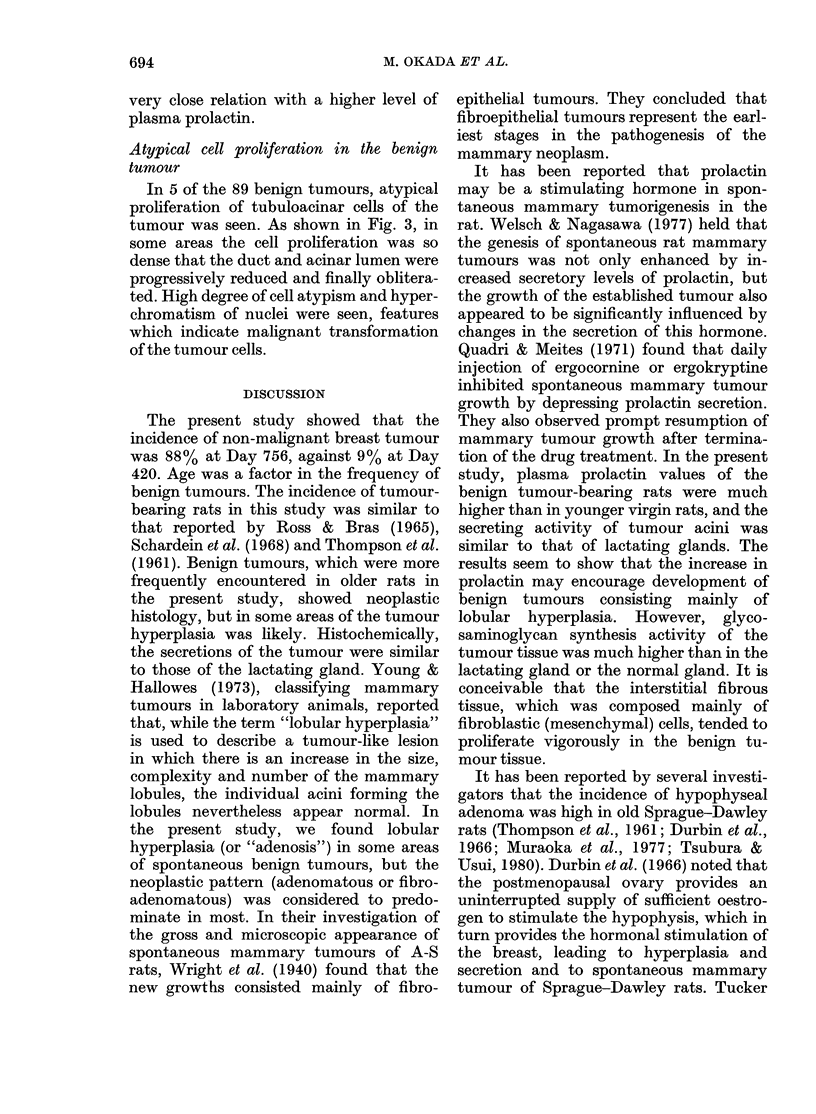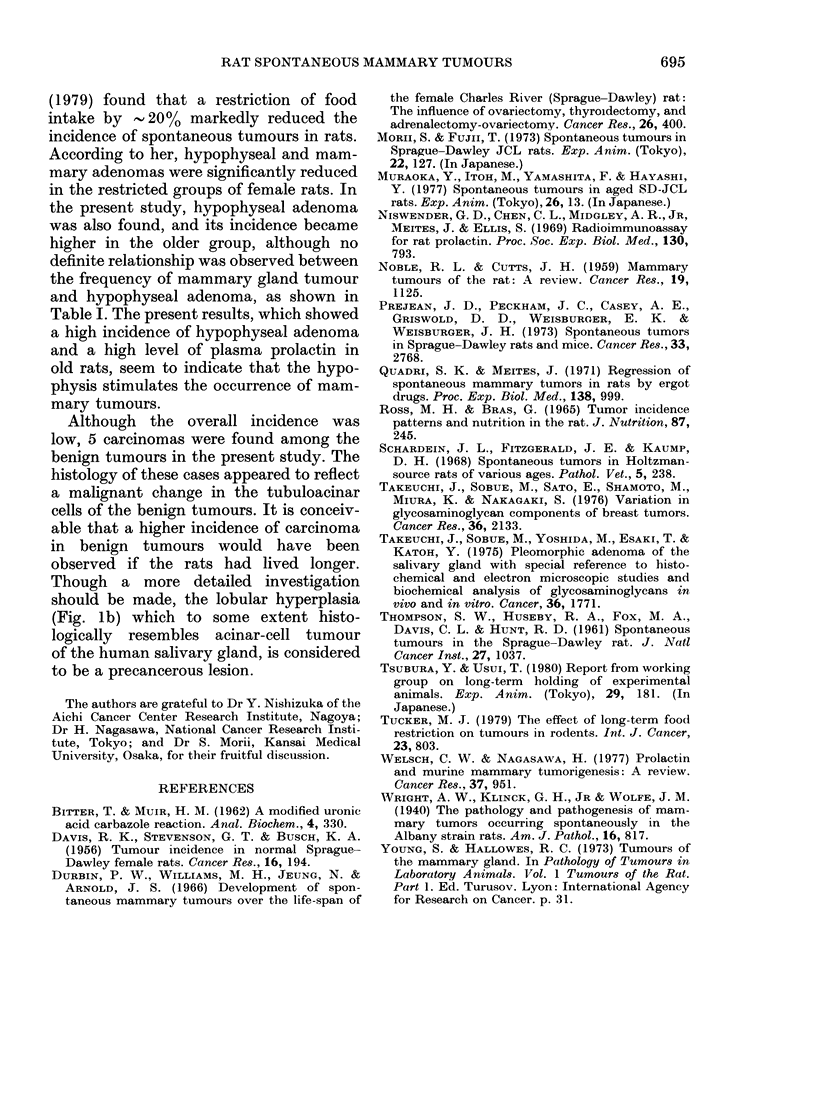# Characteristics of 106 spontaneous mammary tumours appearing in Sprague-Dawley female rats.

**DOI:** 10.1038/bjc.1981.100

**Published:** 1981-05

**Authors:** M. Okada, J. Takeuchi, M. Sobue, K. Kataoka, Y. Inagaki, M. Shigemura, T. Chiba

## Abstract

**Images:**


*Br. J. Cancer* (1981) **43**, 689

### CHARACTERISTICS OF 106 SPONTANEOUS MAMMARY TUMOURS APPEARING IN SPRAGUE–DAWLEY FEMALE RATS

M. OKADA*, J. TAKEUCHI†, M. SOBUE†, K. KATAOKA‡, Y. INAGAKI†, M. SHIGEMURA† AND T. CHIBA*

*From the *Laboratory of Toxicology and Experimental Biology Research, Eisai Co. Ltd, Hajima, Gifu, the †Department of Pathology, School of Medicine, Fujita-Gakuen University, Toyoake, Aichi, and the ‡Department of Internal Medicine, Nagoya University School of Medicine, Nagoya, Japan*

Received 12 September 1980 Accepted 20 January 1981

**Summary.—Pathological studies were undertaken on 106 mammary tumours (89 benign, 17 malignant) appearing spontaneously in 95 normal female Sprague–Dawley rats which were killed at Day 756. The benign tumours comprised those with a predominant acinar hyperplasia and those with adenomatous or fibroadenomatous pattern. No significant differences were found histochemically between the acinar cells of the benign tumours and of the lactating gland, except that the amount of fibrous interstitial connective tissue was larger in the former. $^{3}$H- or $^{35}$S-glycosaminoglycan synthesis by the benign tumours was found to be much higher. The prolactin value in the plasma of the benign-tumour-bearing rats was about 27 times that of 6-month-old virgin rats, and similar to that of rats on the 7th day *post partum*. Carcinomatous proliferation of tubuloacinar cells could be seen in 5 of the 89 benign tumours. The incidence of benign tumours increases with the age of the rats.**

IT IS WELL KNOWN that the incidence of spontaneous tumours in the Sprague–Dawley rat is very high. Noble & Cutts (1959), who reviewed the literature on mammary tumours of the rat, found that, in a group of 150 female Sprague–Dawley rats with an average life span of $760 \pm 21$ days, mammary tumours accounted for 95% of the total tumours found in 54% of the animals. Prejean *et al.* (1973) reported that the percentage of female rats with tumours was almost double that of males. This difference was chiefly attributed to the high incidence of mammary tumours in females, though the largest number of spontaneous tumours occurred in the endocrine system, mainly in the pituitary and adrenal glands of females. Davis *et al.* (1956) classified histologically the spontaneous mammary tumours appearing in normal Sprague–Dawley female rats as adenoma, adenofibroma, fibroma and adenocarcinoma. Morii & Fujii (1973), observing the same kinds of tumours in their laboratory, classified them as fibroadenoma (42·0%), sclerosing adenosis (20·2%), adenoma (10·1%), blunt duct adenosis (7·2%), fibroma (6·1%) and adenocarcinoma (14·5%).

In our laboratory, a high incidence of spontaneous mammary tumours has also been observed in virgin Sprague–Dawley rats. The incidence of benign mammary tumours has been noted to increase with age. In the present study, in order to elucidate the morphological and biological characteristics of the benign mammary tumours, histological and histochemical observation were made, and glycosaminoglycan (GAG) synthesis by the tumour tissues was also investigated to ascertain the physiology of the interstitial element of the tumour. Since a secretory tendency in acinar cells was seen in most of the

Correspondence to: Dr Masaaki Okada, Laboratory of Toxicology and Experimental Biology Research, Eisai Co. Ltd, Hajima, Gifu 483, Japan.

benign tumours, the relationship between plasma prolactin value and tumour appearance was also checked. In several cases, a carcinomatous proliferation of the tubuloacinar cells of the benign tumours was seen.

### MATERIALS AND METHODS

*Animals.* — One hundred and fifty-six female Sprague–Dawley rats (JCL), obtained from Clea Japan Inc., Tokyo, were fed with a standard pellet diet (CA-1, Clea Japan Inc.) and drinking water *ad libitum* in stainless-steel cages without any treatment. Some of them were killed on Day 420, and others on Day 756. The ages, numbers and body weights of the rats killed are shown in Table I. Thirty-two Fischer 344 rats, obtained from Shizuoka Laboratory Animal Center, Hamamatsu, were also used to investigate the spontaneous tumour.

*Histology.*—After death, the rats were necropsied; the subcutaneous solid tumour, if any, lung, liver, spleen, pancreas, endocrine organs, alimentary canal, kidney and brain were excised, fixed in 10% buffered formalin, embedded in paraffin and sectioned. Each of the following dyes were used to stain the sections: haematoxylin and eosin, Alcian blue (pH 2·5), toluidine blue (pH 5·0), PAS, orcein, Mallory–Heidenhain AZAN and Sudan III. In order to detect components of glycosaminoglycans, the digestion test with hyaluronidase (pH 5·0, 100 TRU/ml, 37°C, 1 h), chondroitinase-ABC (pH 8·0, 10 u/ml, 37°C, 1 h) or chondroitinase-AC (pH 7·0, 10 u/ml, 37°C, 1 h) was performed.

*Incorporation of* $^{3}$*H-glucosamine or* $^{35}SO_4$ *into glycosaminoglycans synthesized by spontaneous mammary tumour.*—In order to investigate the physiology of the interstitial element of the tumour tissues, glycosaminoglycan (GAG) synthesis was observed. Immediately after excision, the tumour tissue was cut into thin (1 mm) slices which were incubated in the following medium: 10% dialysed calf serum (Microbial Disease Institute, Osaka University, Osaka) in Eagle's minimal essential medium (GIBCO, Cat. No. F-12) containing 10 $\mu$Ci of $^{35}SO_4$/ml (sp. act. 0·33 Ci/mmol) or 10 $\mu$Ci of $^{3}$H-glucosamine/ml (sp. act. 21 Ci/mmol). After 1 h of incubation at 37°C, the tissue slices were removed and placed in chilled 95% ethanol. Pieces of tissue were washed several times with 80% aqueous ethanol to remove free isotopes, and dried with acetone. After weighing, the resulting dry powder was dissolved in 0·3M NaOH and kept at 4°C overnight. It was then neutralized with 1M HCl, adjusted to pH 8·0 with 1M Tris–HCl buffer, and digested with pronase. The pronase-digested homogenates were centrifuged, and the small amount of insoluble residue without radioactivity was discarded. The supernatant was dialysed against running tapwater overnight and then against 10 volumes of distilled water. GAGs were purified from the supernatant by the procedures described in our previous reports (Takeuchi *et al.*, 1975, 1976).

Analysis of $^{3}$H or $^{35}$S incorporated into each GAG component was performed by cellulose–acetate-membrane electrophoresis. After electrophoresis of the GAG sample, each spot of GAG, stained with Alcian blue, was cut out of the cellulose–acetate membrane, placed in vials and counted. Hexuronic acid was assayed by the carbazole method (Bitter & Muir, 1962) using glucuronic acid as a standard.

The materials used in this study were: chondroitinase-ABC, chondriotinase-AC, hyaluronidase, dermatan sulphate, chondroitin sulphate A and C, and hyaluronic acid from Seikagaku Kogyo Co. Ltd, Tokyo.

*Radioimmunoassay for prolactin.* — Rats were killed by decapitation, and 5 ml of the blood was collected in heparinized tubes. The plasma sample was separated by centrifugation at 4°C, and stored at −20°C until assayed. The rat prolactin reference standard (RP-1), rat prolactin for radioiodination (I-1), and antiserum to rat prolactin (S-2) were obtained from the NIAMDD, U.S.A. The radioimmunoassay method was a modification of the procedure of Niswender *et al.* (1969). Prolactin level in the blood of young female rats before and after parturition was also assayed for comparison.

### RESULTS

### *Mammary-tumour incidence*

The ages, numbers of rats, and mammary-tumour incidence are shown in Table I. In 64 rats killed on Day 420, a total of 13 mammary tumours (6 benign and 7 malignant) were found, whereas a total of 106 tumours (89 benign and 17 malignant) were found in 95 rats at Day

TABLE I.—*Incidence of spontaneous mammary tumours in female Sprague–Dawley rats*

| Group | No. rats | Killed on Day | Body weight mean ± s.e. (g) | Tumour-bearing | Benign tumour | Hypophyseal adenoma* | Carcinoma in benign tumour | Carcinoma |
|---|---|---|---|---|---|---|---|---|
| A | 64 | 420 | 362 ± 16 | 13 | 6 | 2 | 0 | 7 |
| B | 95 | 756 | 529 ± 7 | 72 | 84 | 44 | 5 | 17 |

* Accompanying benign mammary tumour.

756. The malignant tumours were diagnosed as tubular carcinoma. The benign tumours comprised those with predominant acinar hyperplasia and those with adenomatous or fibroadenomatous pattern. The incidence of benign tumours in older rats was much higher than in younger ones. No definite relationship was observed between the frequency of mammary-gland tumour and hypophyseal adenomas.

*Histology of benign tumours*

The benign tumours consisted mainly of acinar and tubular hyperplasia, with the proliferation of the interstitial fibrous connective tissue. The histology of the benign tumours was divided into the following patterns: (a) Hyperplasia of acinar cells, which had an intense secretory activity, with a small fibrous interstitial element (Fig. 1a). This resembled the lactating mammary gland, though the female rats in this study were virgin. (b) A marked proliferation of acinar cells, with scanty fibrous connective tissue. This was similar to the histology of acinar-cell tumour or clear-cell adenoma (Fig. 1b). (c) A marked proliferation of fibrous connective tissue with acinar cell-hyperplasia (Fig. 1c) in some areas, with a histology reminiscent of fibroadenoma. These 3 different types of benign tumour seemed to belong to a single category.

*Histochemical studies of benign tumour for comparison with mammary gland of peri- and postnatal rats*

Intracellular fine granular materials in the acinar cells and homogeneous substance in the tubular lumina of the benign tumour were observed hisotchemically and compared with the lactating mammary gland before and after parturition. As shown in Table II and Fig. 2, no significant differences in the stainability with PAS, Sudan

FIG. 1.—Microscopic sections of benign tumours, showing hyperplasia of acinar cells with (a) intense secretion, (b) adenoma-like pattern, and (c) fibroadenoma-like pattern. H. & E. × 70.

FIG. 2.—Microscopic sections of (a) benign tumour, and (b) lactating mammary gland. Similar stainability is seen in both tissues. PAS. × 200.

TABLE II.—*Stainability of homogeneous substance in the acinar cells and tubular lumina of rat mammary gland*

| | Lactating gland | | |
|---|---|---|---|
| | Perinatal | Postnatal | Benign tumour |
| PAS | + + | + | + + |
| Alcian blue | + + | + | + + |
| Orcein | + | + | + |
| Sudan III | + + | + | + + |

III, Alcian blue or orcein were found between the acinar cells of the benign tumours and the lactating gland. Although the amount of fibrous connective tissue in the stroma was larger in the benign tumour than in the lactating gland, the histochemical findings of acinar cells in both tissues were generally quite similar.

In the interstitial element of the benign tumours, hyaluronate lyase digestion markedly decreased the metachromasia in the stroma, and a large number of metachromatic mast cells, were noticed. The metachromasia in the stroma of the benign tumour was less than in the interstitial constituents of human breast tumours (pericanalicular fibroadenoma), as indicated in our previous report (Takeuchi

TABLE III.—*Radioactivity of* $^{3}H$- *or* $^{35}S$-*glycosaminoglycan and hexuronic acid content in each tissue*

| Rats | Age (days) | Mammary gland | Hexuronic acid ($10^{-3}$ $\mu$mol/mg dry tissue) | Radioactivity incorporated into GAGs (d/min/mg dry tissue) $^{3}H$ | $^{35}S$ |
|---|---|---|---|---|---|
| S.D.* | 756 | Normal | 4·065 | 65·1 | 28·81 |
| S.D. | 756 | Benign tumour | 9·933 | 97·5 | 124·87 |
| S.D. | 756 | Benign tumour | 9·175 | 101·3 | 257·00 |
| Fischer | 458 | Fibroadenoma | 8·658 | 160·6 | 364·39 |

Values show means of 3 pieces of each tissue.
* Sprague–Dawley.

FIG. 3.—Microscopic sections of tumour tissues, showing atypical proliferation of tubuloacinar cells of benign tumours. H. & E. × 200.

*et al.*, 1976). The result shows that hyaluronic acid content is much higher in rat tumours.

*Incorporation of $^{3}H$-glucosamine or $^{35}SO_4$ into glycosaminoglycans by benign tumour tissue*

The GAG-synthesis by benign tumours described above was compared with that of normal mammary-gland tissue of young female rats or fibroadenoma tissues appearing in Fischer 344 rats. $^{3}$H or $^{35}$S radioactivity incorporated into GAGs (ct/min/mg of dry tissue) in each tissue is shown in Table III. The GAG-synthesis in benign tumours, similar to that of fibroadenoma of Fischer 344 rats, is seen to be much higher than in normal mammary-gland tissue. Hexuronic acid content ($10^{-3}$ $\mu$mol/mg dry weight) in benign tumour tissue was also higher than in normal mammary tissue.

*Plasma prolactin values of the tumour-bearing and peri- and postnatal rats*

These values together with those of 6-month-old virgin rats, are shown in Table IV. The prolactin value of tumour-bearing rats were similar to those of rats on the 7th day *post partum*, and about 27 times that of 6-month-old virgins. This result suggests that the occurrence of benign tumours in aged female rats has a

TABLE IV.—*Plasma prolactin values of female rats*

| Rats | No. | Age (days) | Prolactin mean ± s.e. (ng/ml) |
|---|---|---|---|
| Control | 3 | 180 | 18·5 ± 3·2 |
| Benign-tumour-bearing | 7 | 750 | 551·4 ± 141·5 |
| Gestational (days) | | | |
| 13 | 2 | 104 | 30·1 ± 2·0 |
| 14 | 2 | 105 | 27·7 ± 4·1 |
| 15 | 2 | 106 | 25·7 ± 0·7 |
| 16 | 3 | 107 | 40·7 ± 8·3 |
| 17 | 3 | 108 | 36·0 ± 6·9 |
| 18 | 3 | 109 | 19·3 ± 4·4 |
| 19 | 2 | 110 | 19·5 ± 2·7 |
| 20 | 3 | 111 | 82·5 ± 47·9 |
| 21 | 4 | 112 | 122·0 ± 48·1 |
| *Post partum* (days) | | | |
| 7 | 2 | 120 | 414·0 ± 154·0 |
| 14 | 4 | 127 | 282·5 ± 82·4 |
| 21 | 3 | 134 | 127·3 ± 50·9 |

very close relation with a higher level of plasma prolactin.

*Atypical cell proliferation in the benign tumour*

In 5 of the 89 benign tumours, atypical proliferation of tubuloacinar cells of the tumour was seen. As shown in Fig. 3, in some areas the cell proliferation was so dense that the duct and acinar lumen were progressively reduced and finally obliterated. High degree of cell atypism and hyperchromatism of nuclei were seen, features which indicate malignant transformation of the tumour cells.

### DISCUSSION

The present study showed that the incidence of non-malignant breast tumour was 88% at Day 756, against 9% at Day 420. Age was a factor in the frequency of benign tumours. The incidence of tumour-bearing rats in this study was similar to that reported by Ross & Bras (1965), Schardein *et al.* (1968) and Thompson *et al.* (1961). Benign tumours, which were more frequently encountered in older rats in the present study, showed neoplastic histology, but in some areas of the tumour hyperplasia was likely. Histochemically, the secretions of the tumour were similar to those of the lactating gland. Young & Hallowes (1973), classifying mammary tumours in laboratory animals, reported that, while the term "lobular hyperplasia" is used to describe a tumour-like lesion in which there is an increase in the size, complexity and number of the mammary lobules, the individual acini forming the lobules nevertheless appear normal. In the present study, we found lobular hyperplasia (or "adenosis") in some areas of spontaneous benign tumours, but the neoplastic pattern (adenomatous or fibroadenomatous) was considered to predominate in most. In their investigation of the gross and microscopic appearance of spontaneous mammary tumours of A-S rats, Wright *et al.* (1940) found that the new growths consisted mainly of fibroepithelial tumours. They concluded that fibroepithelial tumours represent the earliest stages in the pathogenesis of the mammary neoplasm.

It has been reported that prolactin may be a stimulating hormone in spontaneous mammary tumorigenesis in the rat. Welsch & Nagasawa (1977) held that the genesis of spontaneous rat mammary tumours was not only enhanced by increased secretory levels of prolactin, but the growth of the established tumour also appeared to be significantly influenced by changes in the secretion of this hormone. Quadri & Meites (1971) found that daily injection of ergocornine or ergokryptine inhibited spontaneous mammary tumour growth by depressing prolactin secretion. They also observed prompt resumption of mammary tumour growth after termination of the drug treatment. In the present study, plasma prolactin values of the benign tumour-bearing rats were much higher than in younger virgin rats, and the secreting activity of tumour acini was similar to that of lactating glands. The results seem to show that the increase in prolactin may encourage development of benign tumours consisting mainly of lobular hyperplasia. However, glycosaminoglycan synthesis activity of the tumour tissue was much higher than in the lactating gland or the normal gland. It is conceivable that the interstitial fibrous tissue, which was composed mainly of fibroblastic (mesenchymal) cells, tended to proliferate vigorously in the benign tumour tissue.

It has been reported by several investigators that the incidence of hypophyseal adenoma was high in old Sprague–Dawley rats (Thompson *et al.*, 1961; Durbin *et al.*, 1966; Muraoka *et al.*, 1977; Tsubura & Usui, 1980). Durbin *et al.* (1966) noted that the postmenopausal ovary provides an uninterrupted supply of sufficient oestrogen to stimulate the hypophysis, which in turn provides the hormonal stimulation of the breast, leading to hyperplasia and secretion and to spontaneous mammary tumour of Sprague–Dawley rats. Tucker

(1979) found that a restriction of food intake by ~20% markedly reduced the incidence of spontaneous tumours in rats. According to her, hypophyseal and mammary adenomas were significantly reduced in the restricted groups of female rats. In the present study, hypophyseal adenoma was also found, and its incidence became higher in the older group, although no definite relationship was observed between the frequency of mammary gland tumour and hypophyseal adenoma, as shown in Table I. The present results, which showed a high incidence of hypophyseal adenoma and a high level of plasma prolactin in old rats, seem to indicate that the hypophysis stimulates the occurrence of mammary tumours.

Although the overall incidence was low, 5 carcinomas were found among the benign tumours in the present study. The histology of these cases appeared to reflect a malignant change in the tubuloacinar cells of the benign tumours. It is conceivable that a higher incidence of carcinoma in benign tumours would have been observed if the rats had lived longer. Though a more detailed investigation should be made, the lobular hyperplasia (Fig. 1b) which to some extent histologically resembles acinar-cell tumour of the human salivary gland, is considered to be a precancerous lesion.

The authors are grateful to Dr Y. Nishizuka of the Aichi Cancer Center Research Institute, Nagoya; Dr H. Nagasawa, National Cancer Research Institute, Tokyo; and Dr S. Morii, Kansai Medical University, Osaka, for their fruitful discussion.

### REFERENCES

Bitter, T. & Muir, H. M. (1962) A modified uronic acid carbazole reaction. *Anal. Biochem.*, **4**, 330.

Davis, R. K., Stevenson, G. T. & Busch, K. A. (1956) Tumour incidence in normal Sprague–Dawley female rats. *Cancer Res.*, **16**, 194.

Durbin, P. W., Williams, M. H., Jeung, N. & Arnold, J. S. (1966) Development of spontaneous mammary tumours over the life-span of the female Charles River (Sprague–Dawley) rat: The influence of ovariectomy, thyroidectomy, and adrenalectomy-ovariectomy. *Cancer Res.*, **26**, 400.

Morii, S. & Fujii, T. (1973) Spontaneous tumours in Sprague–Dawley JCL rats. *Exp. Anim.* (Tokyo), **22**, 127. (In Japanese.)

Muraoka, Y., Itoh, M., Yamashita, F. & Hayashi, Y. (1977) Spontaneous tumours in aged SD-JCL rats. *Exp. Anim.* (Tokyo), **26**, 13. (In Japanese.)

Niswender, G. D., Chen, C. L., Midgley, A. R., Jr, Meites, J. & Ellis, S. (1969) Radioimmunoassay for rat prolactin. *Proc. Soc. Exp. Biol. Med.*, **130**, 793.

Noble, R. L. & Cutts, J. H. (1959) Mammary tumours of the rat: A review. *Cancer Res.*, **19**, 1125.

Prejean, J. D., Peckham, J. C., Casey, A. E., Griswold, D. D., Weisburger, E. K. & Weisburger, J. H. (1973) Spontaneous tumors in Sprague–Dawley rats and mice. *Cancer Res.*, **33**, 2768.

Quadri, S. K. & Meites, J. (1971) Regression of spontaneous mammary tumors in rats by ergot drugs. *Proc. Exp. Biol. Med.*, **138**, 999.

Ross, M. H. & Bras, G. (1965) Tumor incidence patterns and nutrition in the rat. *J. Nutrition*, **87**, 245.

Schardein, J. L., Fitzgerald, J. E. & Kaump, D. H. (1968) Spontaneous tumors in Holtzman-source rats of various ages. *Pathol. Vet.*, **5**, 238.

Takeuchi, J., Sobue, M., Sato, E., Shamoto, M., Miura, K. & Nakagaki, S. (1976) Variation in glycosaminoglycan components of breast tumors. *Cancer Res.*, **36**, 2133.

Takeuchi, J., Sobue, M., Yoshida, M., Esaki, T. & Katoh, Y. (1975) Pleomorphic adenoma of the salivary gland with special reference to histochemical and electron microscopic studies and biochemical analysis of glycosaminoglycans *in vivo* and *in vitro*. *Cancer*, **36**, 1771.

Thompson, S. W., Huseby, R. A., Fox, M. A., Davis, C. L. & Hunt, R. D. (1961) Spontaneous tumours in the Sprague–Dawley rat. *J. Natl Cancer Inst.*, **27**, 1037.

Tsubura, Y. & Usui, T. (1980) Report from working group on long-term holding of experimental animals. *Exp. Anim.* (Tokyo), **29**, 181. (In Japanese.)

Tucker, M. J. (1979) The effect of long-term food restriction on tumours in rodents. *Int. J. Cancer*, **23**, 803.

Welsch, C. W. & Nagasawa, H. (1977) Prolactin and murine mammary tumorigenesis: A review. *Cancer Res.*, **37**, 951.

Wright, A. W., Klinck, G. H., Jr & Wolfe, J. M. (1940) The pathology and pathogenesis of mammary tumors occurring spontaneously in the Albany strain rats. *Am. J. Pathol.*, **16**, 817.

Young, S. & Hallowes, R. C. (1973) Tumours of the mammary gland. In *Pathology of Tumours in Laboratory Animals. Vol. 1 Tumours of the Rat. Part* 1. Ed. Turusov. Lyon: International Agency for Research on Cancer. p. 31.